# Immune Defenses of a Beneficial Pest: The Mealworm Beetle, *Tenebrio molitor*

**DOI:** 10.3389/fphys.2019.00138

**Published:** 2019-03-12

**Authors:** Aurélien Vigneron, Charly Jehan, Thierry Rigaud, Yannick Moret

**Affiliations:** ^1^ Department of Epidemiology of Microbial Diseases, Yale School of Public Health, New Haven, CT, United States; ^2^ UMR CNRS 6282 BioGéoSciences, Équipe Écologie Évolutive, Université Bourgogne-Franche Comté, Dijon, France

**Keywords:** *Tenebrio molitor*, innate immunity, pest control, insect farming, ecoimmunology

## Abstract

The mealworm beetle, *Tenebrio molitor*, is currently considered as a pest when infesting stored grains or grain products. However, mealworms are now being promoted as a beneficial insect because their high nutrient content makes them a viable food source and because they are capable of degrading polystyrene and plastic waste. These attributes make *T. molitor* attractive for mass rearing, which may promote disease transmission within the insect colonies. Disease resistance is of paramount importance for both the control and the culture of mealworms, and several biotic and abiotic environmental factors affect the success of their anti-parasitic defenses, both positively and negatively. After providing a detailed description of *T. molitor*’s anti-parasitic defenses, we review the main biotic and abiotic environmental factors that alter their presentation, and we discuss their implications for the purpose of controlling the development and health of this insect.

## Introduction

Insects conflict with human activities due to their ability to successfully colonize and adapt to the majority of terrestrial habitats. They are considered as pests when they damage crops and/or parasitize livestock, which decrease food production, or when they pose a health hazard to humans or other domesticated animals. By contrast, insects are deemed beneficial when they perform valuable services such as pollination and pest control, or when they are bred for being used in human activities. Such a distinction is rather subjective and only arises in light of desired outcomes from a human perspective. In this context, the mealworm beetle, *Tenebrio molitor*, could be seen as either a pest or a beneficial insect. On the one hand, *T. molitor* has generally been considered as a pest because they consume or degrade the quality of stored grains and grain products. On the other hand, mealworms are beneficial, as their larvae are often used as pet food. They also offer a promising alternative protein-rich animal feed and are recommended as a source of human nutrition. Furthermore, *T. molitor* larvae may be further useful due to their ability to efficiently degrade polystyrene and plastic waste (Brandon et al., 2018). For these reasons, *T. molitor* is currently being considered for production at an industrial scale.

*T. molitor* is the host of a wide range of pathogens and parasites such as entomopathogenic microbes, protozoa, and tapeworms, which reduce the mealworm survival or reproductive success. While some of these parasites might be used as biological insecticides to control unwanted population development, they might also be a source of concern in mass rearing facilities. Like other insects, *T. molitor* possesses an arsenal of behavioral, physical, and physiological mechanisms that aim to prevent exogenic invasions or to lower the consequences of a successful infection. The efficacy of these lines of defense may independently or synergistically vary according to biotic and abiotic environmental factors including temperature, food, population density, and individual past interaction with pathogens and parasites. Characterization of such phenotypic plasticity may provide valuable insights for the purpose of improving control or protection of the insect populations. Here we review the prominent *T. molitor* anti-parasitic defense systems and the main environmental factors affecting their presentation. The impact of the environmental factors is considered from the phenotypic to the population level. The processes affecting mealworm anti-parasitic defenses are discussed in the context of controlling the development and health of the insect populations.

## Anti-Parasitic Defenses in the Mealworm Beetle

### Behavioral Immunity

Behavioral immunity refers to altered behaviors used by a host to avoid infection, reduce parasite growth, and/or alleviate disease symptoms. Such anti-parasitic behaviors are increasingly recognized in insects, including *T. molitor*. Behavioral immunity involves anti-parasitic behaviors categorized into three main infectious outcomes (de Roode and Lefèvre, 2012). First, behavioral immunity may provide qualitative resistance by avoiding contact with parasites and pathogens. Behavioral immunity comprises spatial and temporal avoidance of potentially infected places, individuals or food, implementation of hygienic behaviors such as grooming, and adapted social contacts, such as mate choice based on a partner’s immunocompetence. This range of behavior was reported when *T. molitor* were exposed to the tapeworm *Hymenolepis diminuta. H. diminuta* is a rodent parasite that uses the mealworm beetle as an intermediate host. Beetles become infected by consuming eggs of the parasite when feeding on infectious rodent feces. Infection of the beetle is maximized by an increased attractiveness of infected rodents’ feces compared to non-infected feces (Pappas et al., 1995). Infected male beetles, which pay a higher reproductive cost than do infected females (Hurd and Arme, 1986; Hurd and Parry, 1991; Worden et al., 2000), have developed an avoidance behavior for feces that harbor *H. diminuta*, thus decreasing their probability of coming into contact with the tapeworm (Shea, 2010). In addition, females developed qualitative resistance through mate choice, as they are able to evaluate male immunocompetence *via* pheromone signaling and then choose a more immunologically fit mate (Rantala et al., 2002). By choosing a male more refractory to pathogens, females reduce the probability of being infected by their mate and may transmit an enhanced level of immunocompetence to their offspring (Hamilton and Zuk, 1982).

Second, host behaviors may provide quantitative resistance by preventing parasite or pathogen replication. These behaviors involve therapeutic medication, behavioral fever, and grooming, which are particularly beneficial in dense insect populations, where diseases can efficiently spread. No infection outbreak was reported from mealworm mass rearing, but alternatives to antibiotic use could be beneficial to avoid the rise of resistant pathogens. Particularly, adopting therapeutic behavioral medication would be of great interest for the prevention and control of diseases in large populations of beetles. Therapeutic medication can be defined “as a series of behaviors through which infected hosts exploit additional species or compounds to reduce or clear infections, whether mediated through defensive or nutritional properties” (de Roode and Lefèvre, 2012). So far, therapeutic medication has not been reported in the mealworm beetle. Hence, further investigations are needed on this aspect of the mealworm behavioral immunity.

Third, the host may tolerate infections by limiting the negative effects on their reproductive success. This limitation is mainly achieved through increasing their reproductive effort but often at the expense of their longevity. For instance, mealworm beetles tolerate a high number of cysticercoids of the parasite *H. diminuta* at the expense of their own fitness. Nevertheless, in response to parasite infection, males produce improved spermatophores that contain superior nuptial gifts that will be passed to their mating females. This increases female fecundity, and therefore, a higher number of eggs are fertilized by the male (Hurd and Ardin, 2003). Specifically, as beetle longevity is compromised by the parasite, infected males may gain a reproductive benefit, before dying from the infection, by increasing the total protein content of the spermatophores they transfer to females during mating (Carver et al., 1999; Hurd and Ardin, 2003). Males that are experiencing a non-infectious immune-challenge, e.g., a piece of nylon filament inserted into their hemocoel, present a similar increased reproductive effort. Indeed, the immune challenge may induce in males, the perception of a lower survival probability consequent to their simulated infection status, causing the insect to make a last attempt to achieve a maximized level of reproductive success. Consequently, females find those artificially challenged males more sexually attractive, probably due to an increased production of sexual pheromone consequent to the challenge (Sadd et al., 2006; Kivleniece et al., 2010; Krams et al., 2011). The underlying signaling may transit through juvenile hormone (JH), a hormone secreted by the *corpora allata* that is involved in the control of morphogenesis and reproduction in insects, as *T. molitor* males injected with JH are preferred by females (Rantala et al., 2003). So far, comparable adjustment of the reproductive effort upon infection has never been reported in females.

### Cuticle Immunity

The insect integument forms a robust barrier that successfully prevents most parasites and pathogens from colonizing the hemocoel (Moret and Moreau, 2012). It usually constitutes the first barrier between an insect and endogenous invaders. The integument includes an outer layer, called the cuticle, which is produced by a monolayer of epidermal cells. This layer of cells, or epidermis, is separated from the underlying tissues by a thin matrix called the basal lamina. The basal lamina provides a defensive boundary on the insect’s surface due to the thickness of the cuticle and the degrees of sclerotization, or cross-linking, and melanization within cuticular layers. Melanization in the cuticle strengthens its property to act as a physical barrier against the penetration of parasites (Stleger et al., 1988; Hajek and Stleger, 1994). In addition, melanin is toxic to microorganisms and has potent antimicrobial activity (Soderhall and Ajaxon, 1982). Cuticular melanization naturally occurs during the process of molting in insect larvae and nymphs, and right after adult emergence from pupation (Vigneron et al., 2014). However, this process is also induced in response to a mechanical scratch to avoid loss of hemolymph (Benoit et al., 2017) or to microbial invasion (Golkar et al., 1993). In the mealworm beetle, the degree of cuticular melanization is a strong indicator of resistance to the entomopathogenic fungus, *Metarhizium anisopliae*. Indeed, darker beetles are more resistant than lighter ones (Barnes and Siva-Jothy, 2000). This could be explained by the thicker and less porous cuticle displayed by darker insects compared to lighter ones (Evison et al., 2017). A breach in the cuticle also triggers the production of antimicrobial peptides by the epidermal cells, such as cecropins, which are transported in the vicinity of a microbial challenge to abraded cuticle (Brey et al., 1993).

Insect growth and development involve a series of molts during which the old cuticle is partially digested, while a new cuticle is formed and the remnant is discarded. In addition to allowing insect growth, molting may serve as a defense mechanism by reducing the negative effects of a wound or a parasite invasion. For instance, molting quickly in response to a parasite exposure prevents parasites from remaining attached to the cuticle; subsequently, reducing the probability of a successful infection (Duneau and Ebert, 2012; Kim and Roberts, 2012). The benefit of such molting could be exploited by the host inducing precocious molts in response to parasite early attachment (Duneau and Ebert, 2012; Moret and Moreau, 2012). In *T. molitor*, whether wounding or parasite attachment can induce larvae to perform more molts is not known. However, larvae grow through a variable number of molts from 8 to 20. The variability in this number can be partially explained by the availability of resources, the quality of the diet, or the density of the population (Connat et al., 1991; Morales-Ramos et al., 2010). This suggests that the mealworm beetle can adjust its development in response to its environment. Hence, it would be highly relevant to investigate the capacity of the mealworm to molt subsequently to the pressure caused by a wound or a pathogen attempting to invade the insect.

#### The Hemocoelic Immune System

**Figure 1:**
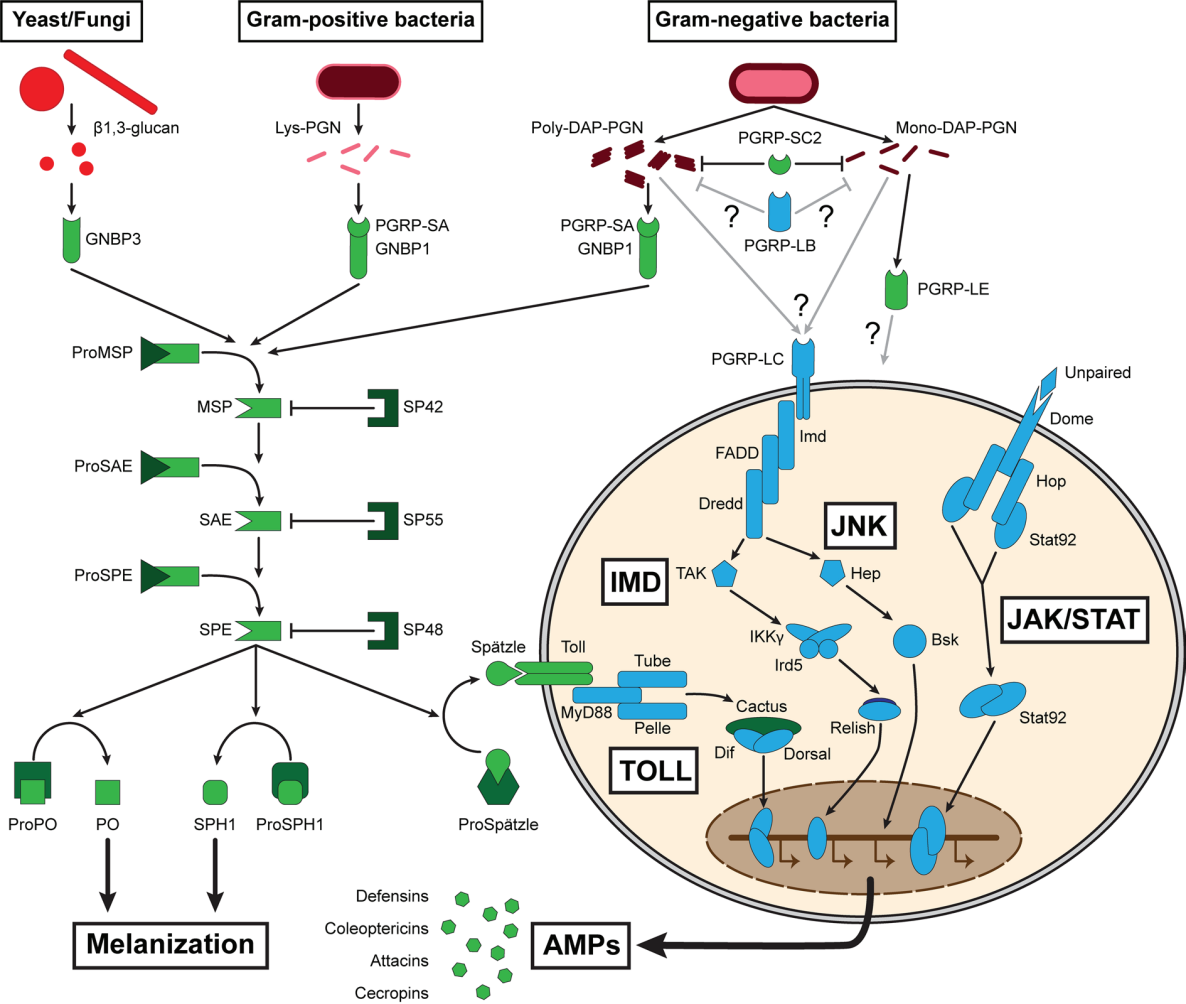
Summary of the major pathways constituting *T. molitor* humoral immune response. Proteins that have been functionally investigated are represented in green, while proteins evidenced at the genomic and/or transcriptomic levels are represented in blue. Lys: lysine; DAP: diaminopimelic acid; PGN: peptidoglycan; PGRP: peptidoglycan recognition protein; GNBP: Gram-negative binding protein; MSP: modular serine protease; SAE: Spätzle-processing enzyme-activating enzyme; SPE: Spätzle-processing enzyme; SP: serine protease; PO: phenoloxidase; MyD88: myeloid differentiation primary response 88; Dif: dorsal-related immunity factor; Imd: immune deficiency; FADD: Fas-associated death domain ortholog; Dredd: death-related ced-3/Nedd2-like protein; TAK: TGF-β activated kinase; IKKγ: inhibitor of nuclear factor-κB kinase subunit gamma, also known as Kenny; Ird5: immune response deficient 5; Hep: hemipterous; Bsk: basket; Dome: domeless; Hop: hopscotch, also known as Jak: Janus kinase; Stat: signal transducer and activator of transcription; AMP: antimicrobial peptide; JNK: c-Jun N-terminal kinase.

Once a parasite or a pathogen has breached the integumental defenses, the insect has to produce a rapid and effective response that localizes and neutralizes the growth and development of the microbe. Like in other insects, hemocoelic defenses of *T. molitor* rely on innate immune effector systems. These involve recognition by pattern recognition receptors (PRRs) that detect a range of conserved non-self microbe-associated molecular patterns (MAMPs), such as bacterial lipopolysaccharides (LPS) and peptidoglycans (PGN), fungal and bacterial β-1,3 glucans, and other sugar moieties. *T. molitor* possesses most known insect PRRs (Johnston et al., 2014). Specifically, these include peptidoglycan recognition proteins (PGRPs) that bind bacterial PGN, Gram-negative binding proteins (GNBPs) that bind LPS, and together with glucan-binding proteins (GBPs), recognizing β-glucans (Figure 1; Zhang et al., 2003; Park et al., 2007; Kim et al., 2008; Lee et al., 2009; Johnston et al., 2014; Yang et al., 2017). Upon recognition, these PRRs trigger the action of various signaling pathways, including the prophenoloxidase cascade regulating melanization processes (Park et al., 2007), the Toll and immune deficiency (IMD) pathways leading to the synthesis of AMPs (Kim et al., 2008; Roh et al., 2009; Yu et al., 2010; Johnston et al., 2014), and hemocyte-driven phagocytosis (Zhu et al., 2013; Kim et al., 2017). Insect innate immune response relies mainly on those pathways, which, *via* their synergic actions, form efficient cellular and humoral responses.

**Figure 2:**
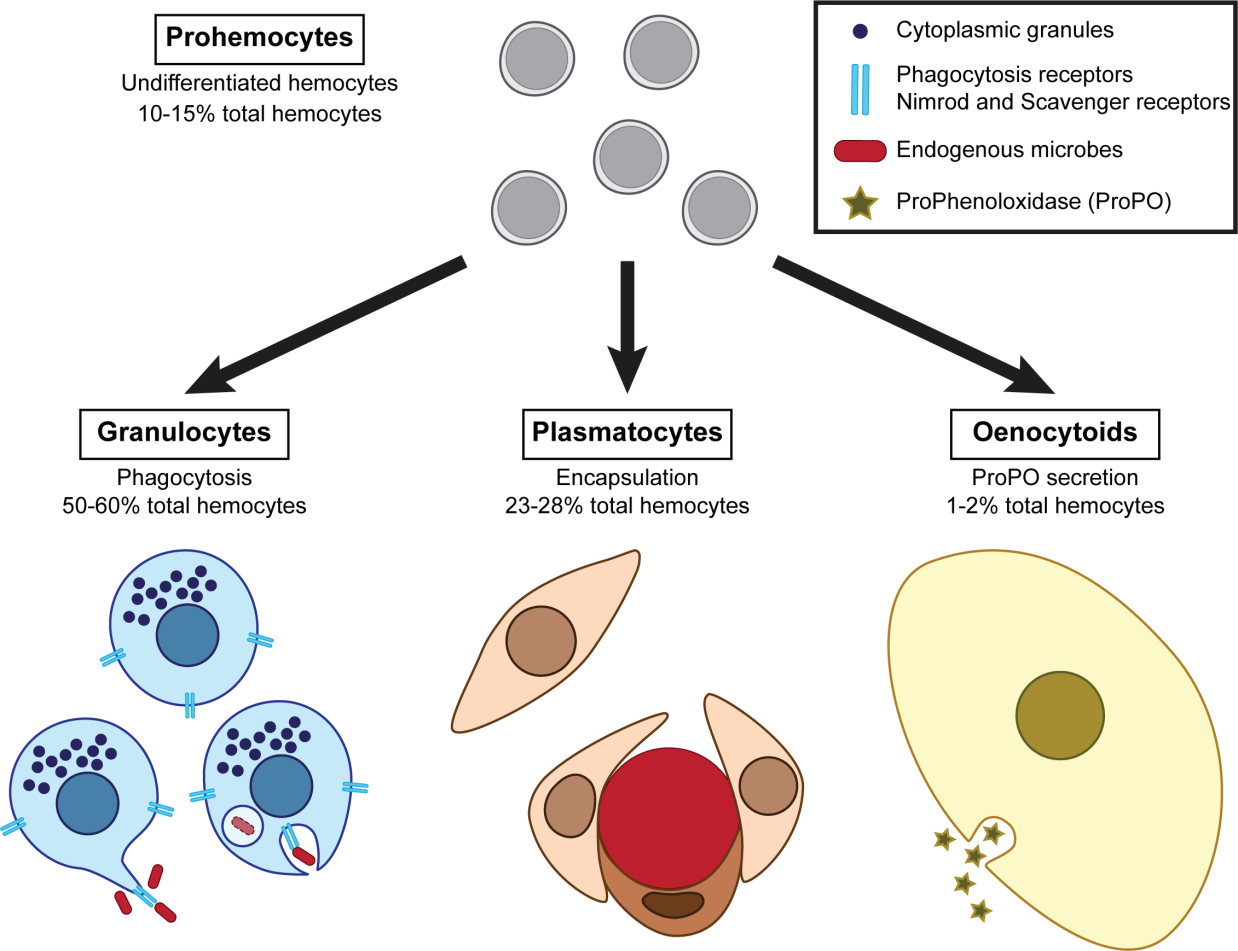
Diversity of the hemocytes composing *T. molitor* cellular immune response and their associated functions.

Cellular defenses primarily involve the action of immune cells called hemocytes, which drive phagocytosis, nodulation, and encapsulation of endogenous organisms. Insects possess several types of circulating hemocytes that are morphologically and functionally distinct, and the prevalence of which is variable in the hemolymph. The mealworm beetle presents four main types of hemocytes: granulocytes, plasmatocytes, oenocytoids, and prohemocytes (Figure 2; Chung and Moon, 2004; Urbanski et al., 2018). Granulocytes account for 50–60% of the observed hemocytes. They are oval cells of about 10 μm in size containing visible dense granules in their cytoplasm and are involved in phagocytosis. Plasmatocytes are the second most abundant hemocytes, representing 23–28% of the total hemocytes. Plasmatocytes are large elongated cells that are likely involved in encapsulation. Oenocytoids are the rarest type as they account for 1–2% of circulating hemocytes. They are large oval cells with a centrally located nucleus and presumably produce enzymes of the melanization cascades. Finally, prohemocytes represent 10–15% of the circulating hemocytes. They are small oval cells less than 10 μm in size with a very large nucleus, probably functioning as precursors of hemocytes.

Hemocyte phagocytosis is achieved upon the recognition of microbes, either directly or after their opsonization by thioester proteins (TEPs), using Scavenger and Nimrod receptors or using the highly variable, alternatively spliced Dscam (Cherry and Silverman, 2006). Particularly, *T. molitor* Scavenger Receptor class C (SR-C) plays a crucial role in the ability of the insect to phagocytose fungi and bacteria (Kim et al., 2017). When an endogenous object is too big to be phagocytized, the cellular immune response also relies on melanization and encapsulation processes. Melanization corresponds to the production of melanin around foreign objects including bacteria, protozoan parasites, nematodes, or parasitoid eggs (Zhu et al., 2013). Upon wounding or recognition of a foreign object by GNBPs and PGRPs, prophenoloxidase (proPO), a zymogen present in some hemocytes and in the plasma, is cleaved through a cascade of serine proteases to liberate the active phenoloxidase (PO). This enzyme catalyzes the production of melanin. In arthropods, levels of melanin and circulating proPO enzymes are used to evaluate immune functions and status. For instance, larvae of the African armyworm, *Spodoptera exempta*, reared at high densities exhibit higher proPO levels in the hemolymph and higher resistance to nucleopolyhedroviruses than those reared solitarily (Reeson et al., 1998; Wilson et al., 2001). In addition, parasitized bumblebees (*Bombus terrestris*) exhibit twice as much PO activity in their hemolymph than do non-parasitized nest mates (Brown et al., 2003). *T. molitor* melanization processes play an important role in the ecology of the insect, as cuticle darkness polymorphisms correlate with PO activity, with a darker cuticle meaning a higher PO activity (Armitage and Siva-Jothy, 2005). The plasticity of those traits could indicate a higher cost for the insect to maintain a more efficient immune system (Barnes and Siva-Jothy, 2000). This could be due to the fact that the enzymatic cascade leading to melanization is accompanied by the production of cytotoxic intermediates, such as phenols, quinones, and reactive oxygen species (Nappi and Vass, 1993; Nappi and Ottaviani, 2000; Sugumaran et al., 2000; Nappi and Christensen, 2005), which help to kill invading organisms, but at the cost of deleterious effects for the host (Sadd and Siva-Jothy, 2006). However, fecundity and lifespan of darker insects reared in laboratory conditions were not impacted (Krams et al., 2016). Unidentified trade-offs structuring insect life history traits may prevent the fixation of the darker cuticle phenotype in the wild.

Those intermediates are also part of a more systemic immune response as they are liberated in the insect hemolymph along with the inducible synthesis of AMPs produced by the fat body. This response constitutes the humoral immune defense, which is triggered upon microbe recognition *via* the Toll and IMD signal transduction cascades, complemented by c-Jun N-terminal kinase (JNK), and Janus kinase/Signal Transducer and Activator of Transcription (JAK/STAT) pathways (Figure 1; Buchon et al., 2014). These pathways activate the NF-κB transcription factors Relish, Dorsal, and Dif, which induce expression of antimicrobial peptides (Kounatidis and Ligoxygakis, 2012). These pathways are conserved in many insects including *T. molitor* (Johnston et al., 2014). Contrary to the transient and immediate induction of cellular immune effectors, *T. molitor* antimicrobial production is induced within 48 h following MAMP detection and last at least 14 days (Haine et al., 2008; Johnston et al., 2014). The mealworm beetle presents several genes potentially coding for antimicrobial peptides, including the Tenecins 1, 2, 3, and 4, for which the proteins have been isolated and assessed for their immune functions (Moon et al., 1994; Lee et al., 1996; Kim et al., 1998; Roh et al., 2009; Park et al., 2010b; Chae et al., 2012). Tenecins 1, 2, and 4 are inducible defensin, coleoptericin, and attacin, respectively, which are regulated by the Toll and IMD pathways, and mainly display antibacterial activities (Kim et al., 1998; Kim et al., 2001; Roh et al., 2009; Park et al., 2010b; Chae et al., 2012). Tenecin 3 is a constitutively expressed Thaumatin, the role of which is suggested to prevent fungal infections (Chae et al., 2012; Maistrou et al., 2018). Interestingly, AMPs are induced in *T. molitor* eggs in response to a septic injury (Jacobs et al., 2017). Moreover, the levels of immune gene expression in the eggs reach comparable levels to the expression in adults. This suggests that eggs contain immunocompetent cells in addition to maternal effects to defend themselves against potential invaders. Investigation of egg immunity in *Tribolium castaneum* has shown that most of the immune response originates from the serosa, an extra layer of cells that envelops the yolk and the developing embryo (Jacobs et al., 2014). It is not known if *T. molitor* serosa is capable of such immune response, but this is likely due to their close relation to *T. castaneum* and the similar environment they exploit.

## Source of Variation in Anti-Parasitic Defenses

### Density

The risk for an individual to be infected with pathogens and parasites increases when it lives in a population with higher density. Hence, it would be beneficial for individuals developing in such conditions to invest more in their defense mechanisms than individuals experiencing a low-density environment. This hypothesis was introduced as “density-dependent prophylaxis” (DDP) and predicts that individuals developing in high-density conditions will exhibit a more efficient immune response against parasites and pathogens (Wilson and Reeson, 1998). This concept was first defined investigating the noctuid moth *Spodoptera exempta*, which presents a higher resistance to baculovirus when developing in high-density conditions (Wilson and Reeson, 1998). These density-dependent effects are associated with elevated antibacterial activity and higher numbers of circulating hemocytes in the hemolymph (Reeson et al., 1998; Wilson and Reeson, 1998). DDP is observed in several insects, including both holometabolous insects (Cotter et al., 2004a,b) such as *T. molitor* (Barnes and Siva-Jothy, 2000) and hemimetabolous insects (Wilson et al., 2002), suggesting that it is widely conserved among distant species and even in other invertebrates (Mills, 2012).

**Figure 3:**
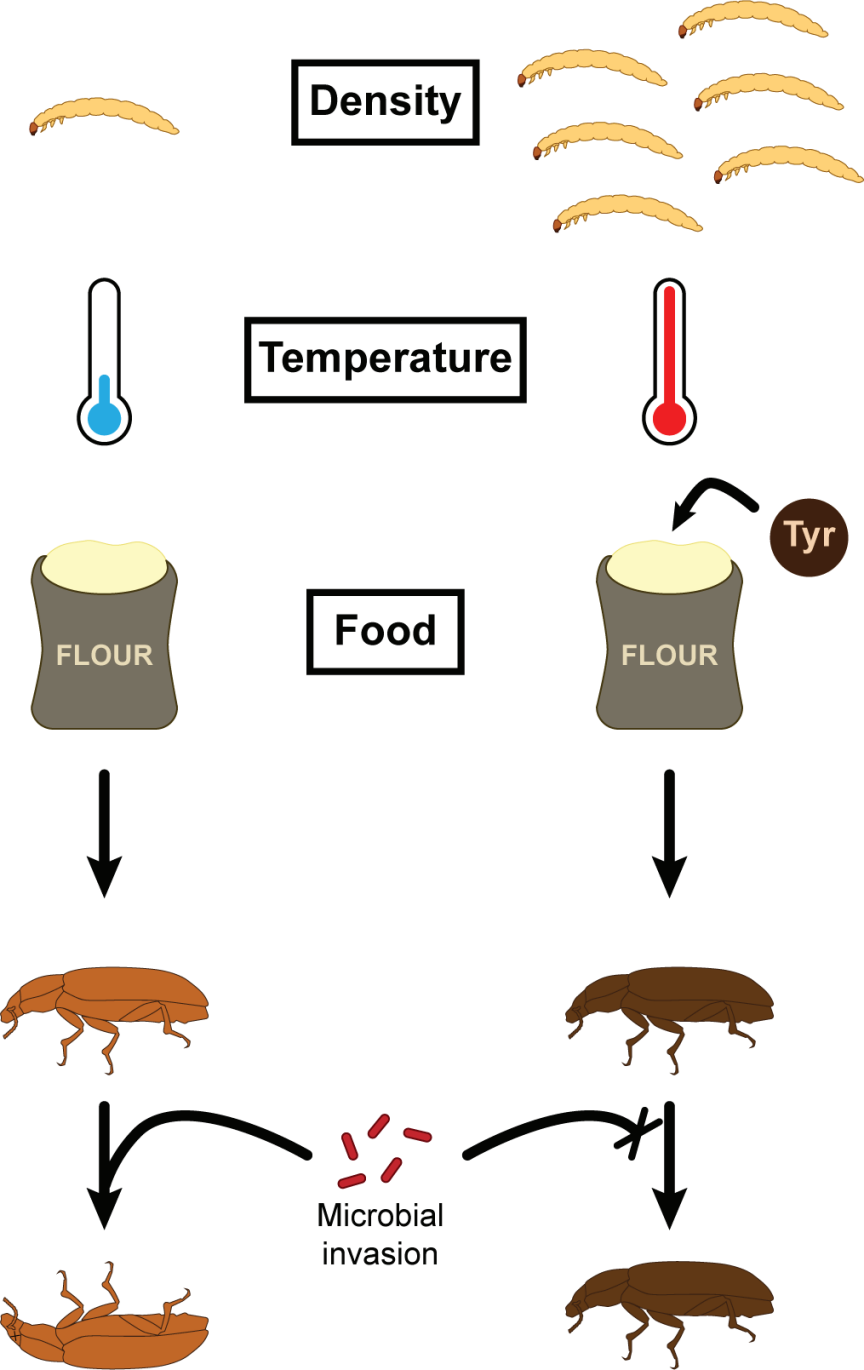
Factors influencing *T. molitor* cuticle color and subsequent immune defenses.

Interestingly, DDP usually correlates with melanism polyphenism, for which insects living in high-density population are darker (Figure 3; Barnes and Siva-Jothy, 2000; Wilson et al., 2001; Wilson et al., 2002; Cotter et al., 2004a). This degree of melanization is the main factor correlating with higher resistance to microbial invaders (Barnes and Siva-Jothy, 2000; Cotter et al., 2004a). Hence, it can be argued that the DDP-driven improved resistance to pathogens comes from the higher number of darker individuals generated in high-density conditions rather than being a shared trend among insect reared in high-density conditions, independently of their color (Barnes and Siva-Jothy, 2000; Cotter et al., 2004a). This is especially supported in *T. molitor* as darker individuals, compared to paler insects, present a thicker and less porous exocuticle (Silva et al., 2016) and an increased PO activity (Evison et al., 2017) and do not suffer the same deleterious effects following a mock hemolymph infection (Krams et al., 2016) independently of the population density. Nevertheless, the plasticity of the melanization phenotypes in response to population density added to the absence of predominance of darker individuals among *T. molitor* populations suggests underlying trade-offs preventing the fixation of the darker phenotype.

While population density influences insect melanization, the underlying molecular mechanisms governing DDP are still unclear. Nevertheless, DDP demonstrates that interaction between individuals can structure their development, including the immune defenses. Horizontally transferred immunity from insects exposed to an infection to naïve insects is another aspect of individual interaction shaping the efficiency of immune defenses. Social and behavioral immunity have been mainly described in eusocial insects (Traniello et al., 2002; Ugelvig and Cremer, 2007) and may be relevant in highly interacted insect populations (Elliot and Hart, 2010). A recent study investigated whether *T. molitor* displays social immunity through exposing *Staphylococcus aureus*-infected insects to naïve individuals (Gallagher et al., 2018). The authors did not report any evidence leading to the conclusion that social immunization exists in the mealworm beetle. However, they observed an improved tolerance to bacterial infection in naïve insects that were interacting with insects injected with heat-killed bacteria (Gallagher et al., 2018). This suggests that *T. molitor* can sense and respond to immune-related signals originating from pathogen-exposed individual.

Density- and infection-sensing can also modulate resistance mechanisms across generations, as shown by the water flea, *Daphnia magna* (Michel and Hall, 2016), and the cotton leafworm, *Spodoptera littoralis* (Wilson and Graham, 2015). In those models, crowding the parental generation conditioned the immune response of the offspring associated to increased levels of parasite resistance. No such result has been yet demonstrated in *T. molitor*. This insect can protect the egg through the transfer of maternal immune effectors and the induction of egg immune genes in response to a parental microbial challenge (Dhinaut et al., 2018b). Hence, because the mealworm displays DDP, it would be highly relevant to investigate whether high-density conditions could lead to improve egg immunity.

### Temperature

With the rising concern for climate change, a growing number of studies have focused on how temperature fluctuation impacts insect biology. One recent aspect of this field of investigations relates to the influence of temperature on insect immunity. Temperature stress triggers the production of heat-shock protein (HSP) that helps organisms to sustain said stress. Interestingly, those proteins are also expressed in *T. castaneum* following a microbial infection (Altincicek et al., 2008). Another example comes from *Musca domestica* that is expressing Hsp70 in response to a bacterial stimulation, and for which the knockdown *via* RNA interference (RNAi) leads to a decrease resistance to infection (Tang et al., 2012). Altogether, these results show that temperature-related genes are intimately intricate with immune pathways, suggesting a potential influence of temperature on immune defenses.

Several recent studies have investigated the effects of temperature on life history traits of insects and conclusions vary depending on the model studied (Prokkola et al., 2013; Kaunisto et al., 2015; Silva and Elliot, 2016; Yin et al., 2016; Laughton et al., 2017). For example, when reared in a higher set of temperatures, the Indian meal moth, *Plodia punctella*, presents more hemocytes (Laughton et al., 2017). Conversely, the velvet bean caterpillar, *Anticarsia gemmatalis*, presents fewer hemocytes when developing at a higher temperature (Silva and Elliot, 2016). As organisms evolve and build trade-offs between their life history traits influenced by their environment, it is expected that mechanisms underlying resistance or tolerance to stress correlate differently to the impacted life history traits. It is especially relevant for insects, which, because they are ectothermic, must resist the variations of their environmental temperature. In *T. molitor*, investigations have shown that encapsulation and cuticle darkness negatively and positively correlate with temperature, respectively (Figure 3; Prokkola et al., 2013). Moreover, while developing in warmer temperature conditions, the mealworm beetle experiences a shortened larval development and presents longer elytra. It shows that the insect biology in its whole is impacted by the temperature, which imposes trade-offs between different life history traits of an individual.

While temperature stress impacts insect immune defenses, little is known about how and whether it also affects the immune system of offspring. This inquiry has been explored in *T. castaneum*, where the offspring resistance to a bacterial challenge was found to be improved when both parents received a cold shock, while no effects were observed when the parents received a heat shock (Eggert et al., 2015). In addition, a cold shock experienced by the mother or both parents induced higher PO activity in the offspring, while a heat shock of either parent, or both, reduced the PO activity of their offspring. In conclusion, temperature is an important parameter that can significantly influence the biology of individuals, including their immune system. Hence, it would be beneficial to further investigate how temperature relates to *T. molitor* in the aim to better promote or regulate its population.

### Food

Food quality and quantity are critical to immune defenses against parasites and pathogens. While leveraging food amount and quality for restricting or controlling pest populations is difficult, we may use nutrients that directly or indirectly improve the immune system of insects that we would like to maintain or mass rear.

As immune functions require metabolic resources, food restriction can impair immune activity. For instance, adult *T. molitor* PO activity can be reduced by half during short-term food privation, but it returns rapidly to initial levels when given access to food again (Siva-Jothy and Thompson, 2002). Furthermore, following an immune challenge, *T. molitor* larvae can eat five times more food per day than usual to compensate for the caloric expense of the immune response (Catalan et al., 2011). Hence, unsurprisingly, food supply is important to keep insects healthy. While the amount of food available matters, its nutritional composition is also important, especially with regard to its protein to carbohydrate ratio (Ponton et al., 2011). For instance, infected caterpillars select food containing higher protein to carbohydrate ratio, which improves their resistance to viral or bacterial infection (Lee et al., 2006; Povey et al., 2009). The same observation has been made for *T. molitor,* whose healthy larvae usually prefer diets with lower protein to carbohydrate ratio but shift toward food with higher protein contents after immune challenge with bacteria (Catalan et al., 2011). As a consequence of this diet shift, hemocyte circulation and antibacterial activity are enhanced in the hemolymph, which presumably maximizes resistance against bacterial infection. However, PO activity is not affected by this shift in diet-choice, suggesting that either PO activity is likely less sensitive to protein intake, or it is limited by a trade-off consequent to the upregulation of antibacterial activity (Moret and Schmid-Hempel, 2001; Cotter et al., 2004b; Moret and Schmid-Hempel, 2009). In fact, diet effects on PO activity appear controversial in insects, as it has been found to be slightly enhanced by diets rich in either proteins (Lee et al., 2006; Povey et al., 2009) or carbohydrates (Koella and Sorensen, 2002; Cotter et al., 2011). Nevertheless, PO activity appears less variable with respect to diet than other immune traits (Catalan et al., 2011; Cotter et al., 2011; Evison et al., 2017). On the one hand, excess levels of PO activity could be dangerous, as uncontrolled activation of PO in the hemocoel would result in the production of toxic quinones and reactive oxygen species, which could harm self-tissues and organs (Nappi and Vass, 1993; Sadd and Siva-Jothy, 2006). Therefore, preventing excessive diet-mediated upregulation of PO activity might be required. On the other hand, PO is also involved in a large set of physiological functions independent of immunity (Hiruma and Riddiford, 1988). Thus, preventing its excessive downregulation may help to maintain homeostasis of those other physiological functions.

Interestingly, immune-challenged *T. molitor* larvae exhibit significant weight loss when fed either protein- or carbohydrate-rich diets, while their weight remains stable when they were given both protein- and carbohydrate-rich diets (Catalan et al., 2011). This suggests that no single blend of ingested nutrients can optimize all the physiological needs, and that the composition of the diets ingested by the insects represents a trade-off between optimizing different traits (Cotter et al., 2011). Hence, a relevant way to maximize growth and immunity of mealworm beetles would be to supply the insect with nutrient adjusted according to their physiological needs.

While diet composition appears to exert a moderate effect on hemocoelic PO activity, it may nevertheless influence cuticle melanization and sclerotization, which subsequently impacts cuticular color (Reeson et al., 1998; Wilson et al., 2001; Cotter et al., 2004a) and resistance to important entomopathogens (Wilson and Reeson, 1998; Barnes and Siva-Jothy, 2000; Wilson et al., 2001; Dubovskiy et al., 2013). PO enzymes mediate melanization and sclerotization of the cuticle (Andersen, 2010), and central to these latter processes is the production of 3, 4-dihydroxyphenylalanine (DOPA) from the hydroxylation of the semi-essential amino acid tyrosine (Vavricka et al., 2010). *T. molitor* exhibits plastic variation in cuticle color, and darker individuals are often more resistant to fungal diseases (Barnes and Siva-Jothy, 2000). Darker individuals exhibit alteration in the physical and chemical properties of the cuticle such as thickening and a higher degree of melanization (Silva et al., 2016; Evison et al., 2017). The experimental supplementation of *T. molitor* larvae with the amino acid Tyrosine led to the development of adults with a darker cuticle (Figure 3; Evison et al., 2017). Interestingly, the cuticle was also thicker, but only in females, suggesting that males and females are allocating their tyrosine resources differently. In addition, while tyrosine supplementation resulted in improved cuticle defenses, it did not affect hemocoelic melanin-mediated defenses (Evison et al., 2017), suggesting that the regulation of both lines of defense could be uncoupled despite their similar precursor.

**Figure 4:**
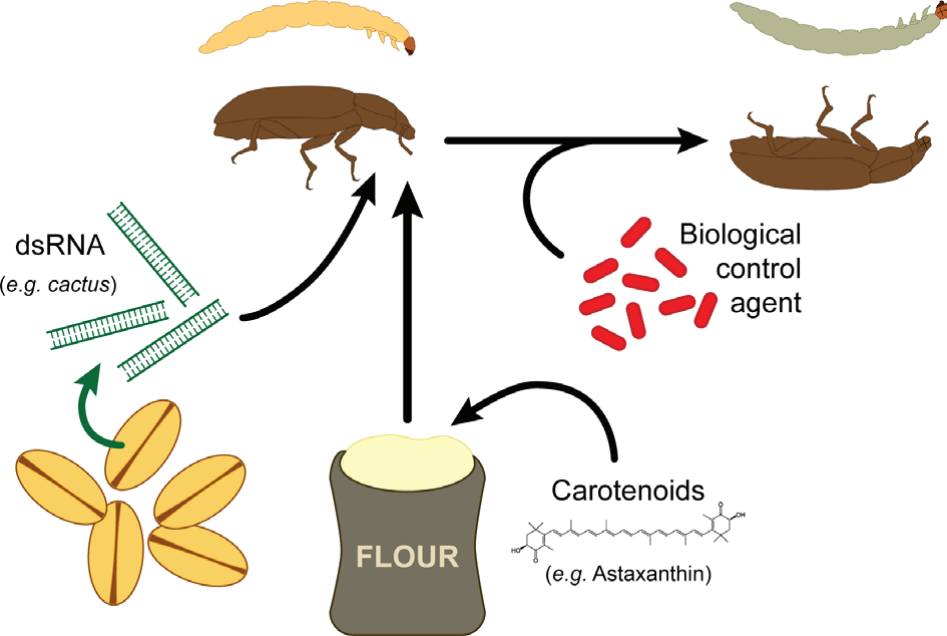
Potential methods to weaken *T. molitor* immune system with the aim of enhancing population control using biological agents.

Insect immune activities are associated with the production and release of cytotoxic compounds such as reactive oxygen and nitrogen species (ROS and RNS, respectively) (Nappi and Vass, 1993). While these toxic substances help to kill invading organisms, they also cause self-damage in *T. molitor* (Sadd and Siva-Jothy, 2006), which results in a significant lifespan reduction (Pursall and Rolff, 2011; Khan et al., 2017). While insects rely on endogenous antioxidants to scavenge these free radicals, this process might be supported by dietary sources of antioxidants (Chew and Park, 2004). For instance, carotenoids have the ability to scavenge free radicals produced by immune activities (El-Agamey et al., 2004) and the potential to interact with endogenous antioxidant enzymes (Lee et al., 2011; Babin et al., 2015). Importantly, in addition to their potent antioxidant property, carotenoids stimulate the immune system of both vertebrates (Blount et al., 2003; Park et al., 2010a) and invertebrates (Flores et al., 2007; Babin et al., 2010; Babin et al., 2015). Contrary to these general observations, life-time dietary supplementation of *T. molitor* with astaxanthin, a carotenoid with strong antioxidant activity (Chew and Park, 2004), strongly depressed the insect immune system and decreased its resistance to bacterial infection (Dhinaut et al., 2017). Investigations pointed that this could result from the interaction between the pigment and nitric oxide (NO), which stimulates both cellular and humoral immunity of insects (Imamura et al., 2002; Kraaijeveld et al., 2011; Eleftherianos et al., 2014; Sanzhaeva et al., 2016). Indeed, astaxanthin may either inhibit the activity of the nitric oxide synthase, the enzyme responsible of NO production from L-Arginine (Hussein et al., 2006), or interfere with NO cellular signaling by scavenging a fraction of circulating NO, and consequently, downregulating base levels of immune activities. In addition, astaxanthin may also have regulatory effects on the host’s metabolism, which would collaterally impair immune activities (Hussein et al., 2007; Yang et al., 2011). Astaxanthin interacts with nuclear receptors of the peroxisome proliferator-activated receptor superfamily, which regulates lipid and glucose metabolism in vertebrate (Jia et al., 2011). Such an alteration of the host metabolism may reduce the allocation of energetic resources to the immune system. If these receptors are conserved among taxa, similar regulatory effects may also occur in insects. These results suggest that supplying *T. molitor* with carotenoids, especially with astaxanthin, is rather detrimental. Hence, the use of this carotenoid might not be adequate when rearing this insect. By contrast, its immune-depressive effect could be used to improve the success of microbial insecticides, where the insect is detrimental (Figure 4).

### Previous Experience of Pathogens: Immune Priming

Like other invertebrates, *T. molitor* lacks immune effectors that are responsible for the acquired immune response of vertebrates. However, the invertebrate immune system is capable of functional modulation similar to the acquired immune response of vertebrates (Moret and Siva-Jothy, 2003; Moret, 2006). This form of innate immune memory in an invertebrate is termed “immune priming,” which is broadly defined as increased protection to a pathogen following previous exposure (Little and Kraaijeveld, 2004). Immune priming may exhibit a variable degree of specificity, from cross-reactive (non-specific) (Moret and Siva-Jothy, 2003) to highly specific (more effective against the pathogen encountered during the primary challenge), especially when cellular processes are involved for the latter (Pham et al., 2007; Roth and Kurtz, 2009). Priming response can also be obtained from a challenge with an inert immune elicitor such as a nylon implant, which can provide immune protection against a subsequent fungal infection (Krams et al., 2013). This suggests that immune priming originates, at least partially, from activation of immune defenses rather than solely from the presence of MAMPs.

Functionally, individual immune priming may rely on three types of responses (Coustau et al., 2016). First, it may involve a sustained response, corresponding to the long-lasting upregulation of the same immune effectors after the initial immune challenge. Second, a recalled response results in a faster and stronger response after a secondary infection in a way that is reminiscent of the vertebrate acquired immune response. Third, priming may induce an immune shift, involving different immune effector systems during the primary and the secondary immune responses. Current evidence suggests that individual immune priming in *T. molitor* is achieved through a sustained antibacterial activity, which can be active for at least 20 days after a primary immune challenge by injection of a suspension of killed Gram-positive bacteria (Makarova et al., 2016; Dhinaut et al., 2018a). Hemocyte concentration also increased two-fold upon the secondary challenge with the bacteria (Dhinaut et al., 2018a), which might be consistent with a hemocyte-mediated recall response. However, while this enhanced hemocyte concentration could be involved in the priming response, this relationship is speculative because of the persistent antibacterial activity in the hemolymph resulting from the primary challenge (Dhinaut et al., 2018a). No such long-lasting antibacterial activity and change in hemocyte concentration was found in primed insects with Gram-negative bacteria, and consistently, the priming with Gram-positive bacteria provided the most effective protection against microbial reinfection (Dhinaut et al., 2018a). Therefore, individual priming responses induced by Gram-positive bacteria are stronger and more protective than those induced by Gram-negative bacteria in *T. molitor*, possibly because Gram-positive bacteria have played an important evolutionary role in shaping the immune system of this insect.

**Figure 5:**
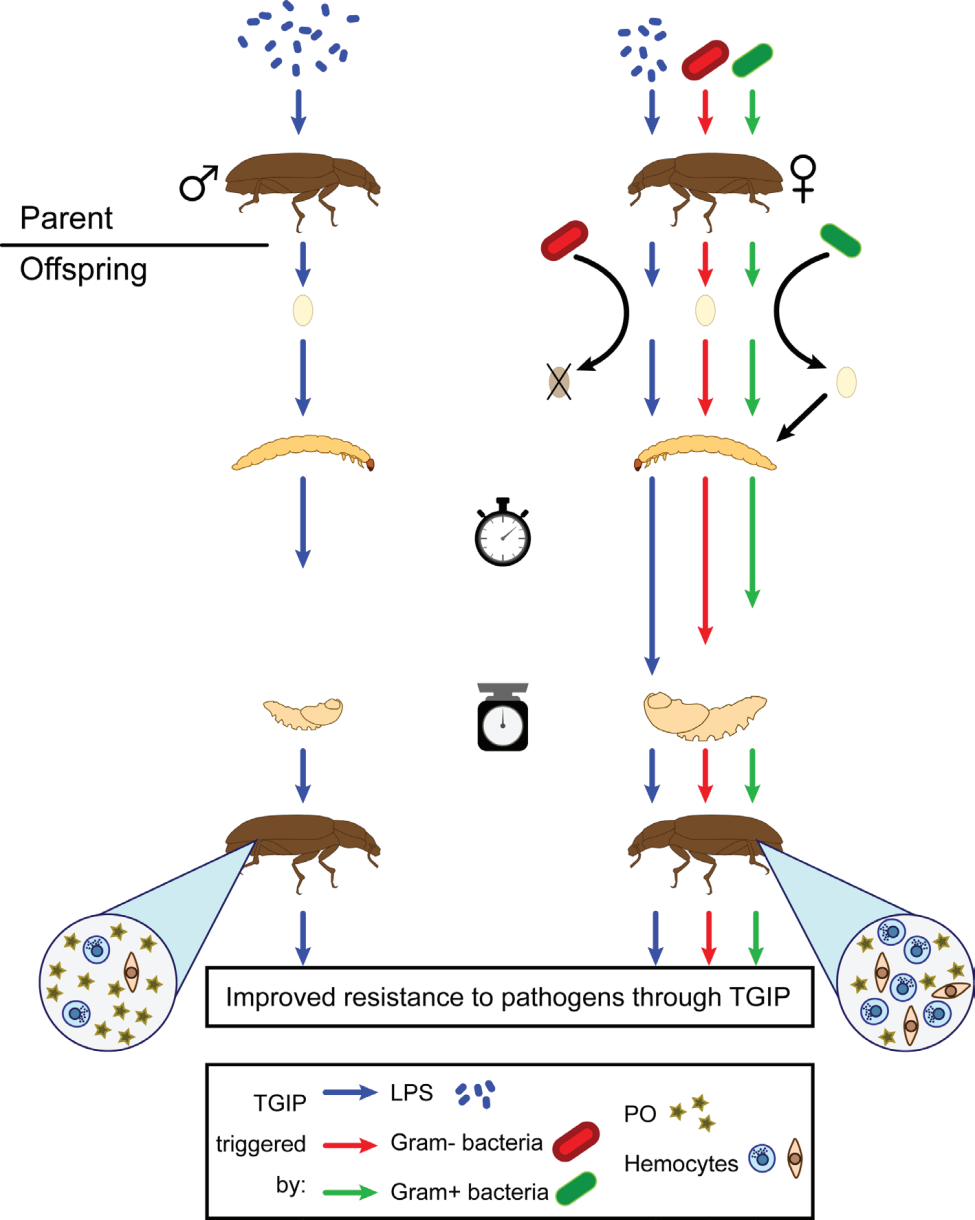
Subsequent effects of TGIP on *T. molitor* offspring traits. TGIP enhances offspring resistance to pathogens if either the father (left) or the mother (right) has been primed. However, the sex of the parent triggering the TGIP produces different consequences on the offspring traits. Offspring originated from a mother primed with either LPS (blue), Gram-negative bacteria (red), or Gram-positive bacteria (green) are also affected differently by the TGIP. Larval developmental time (stopwatch) is represented by the length of the arrows between larvae and pupae (arbitrary scale). Weight of the pupae (scale) is represented by the size of the scheme at this stage (arbitrary scale). Density of hemocytes and hemolymph phenoloxidase (PO) activity is represented by the relative abundance of their corresponding symbols in the circles originated from the offspring beetles.

An individual may not only gain immune protection from its own immunological experience, but it can also benefit from that of its parents through “trans-generational immune priming” (TGIP). TGIP allows immune-challenged parents to produce more resistant offspring (Figure 5; Moret, 2006). In *T. molitor*, TGIP effects were revealed through enhanced immune activity in primed eggs (Moreau et al., 2012; Zanchi et al., 2012; Dubuffet et al., 2015; Dhinaut et al., 2018b), larvae (Moret, 2006), and adult offspring (Zanchi et al., 2011; Dhinaut et al., 2018b). Furthermore, enhanced immunity in the offspring may result either from the immune challenge of fathers or mothers, although paternal and maternal TGIP are associated with the enhancement of different immune effectors in the offspring (Zanchi et al., 2011). Similar to individual immune priming, TGIP of offspring does not appear to be pathogen-specific (Dhinaut et al., 2018b). For example, the offspring of mothers primed with the Gram-negative bacteria, *Serratia entomophila*, and those primed with the Gram-positive bacteria, *Bacillus thuringiensis,* had a similar enhanced survival to bacterial infection (Dhinaut et al., 2018b). However, while the maternal challenge with *S. entomophila* had no apparent effect on base levels of cellular or humoral immune defenses of the offspring, the maternal challenge with *B. thuringiensis* slightly enhanced PO activity of the offspring (Dhinaut et al., 2018a). In addition, the offspring of mothers immunized with purified LPS from bacterial cell wall displayed increased base levels of hemocyte concentration (Zanchi et al., 2011). Hence, offspring immunity is affected differently through TGIP depending on the nature of the maternal challenge.

Although TGIP occurs in several invertebrate species, investigations on its molecular mechanisms have just begun. In *T. castaneum*, bacterial components cross the midgut epithelium and are stored in eggs (Knorr et al., 2015), probably *via* a vitellogenin-mediated transfer. Vitellogenin, the major egg storage resource for embryo nutrition, also displays multiple immune functions by acting as a multivalent pattern recognition receptor with opsonin and antibacterial activity (Singh et al., 2013). Vitellogenin can recognize bacteria by specifically binding to MAMPs, such as PGN and LPS, and mediate the translocation of bacterial proteins to the eggs (Salmela et al., 2015). This transfer was associated with an increased expression of immune genes in the eggs (Knorr et al., 2015; Salmela et al., 2015). Interestingly, *T. molitor* females provide enhanced antibacterial activity to eggs that are produced from the second to the eighth day after the maternal challenge only (Zanchi et al., 2012). Offspring resulting from eggs laid after this restricted period of time are not protected by enhanced antibacterial activity, but they still exhibit a higher concentration of hemocytes in their hemolymph at the adult stage (Zanchi et al., 2011). These results suggest that mechanisms regulating protection of eggs and adult offspring are probably different. Hence, TGIP likely involves independent mechanisms that are acting simultaneously or sequentially over the development of the insect.

How and where the signal of a primary infection is “memorized” appears of paramount importance in the understanding of within and trans-generational immune priming. While hemocytes may play a role in immune memory, evidence is still scarce. For instance, during the antiviral immune response of *Drosophila*, infected cells generate double-stranded RNA (dsRNA) to inhibit viral molecule expression *via* RNAi (Tassetto et al., 2017). Part of the produced dsRNA is taken up by hemocytes, which then produce virus-derived complementary DNAs (vDNA) used as templates for *de novo* synthesis of small interference RNAs (siRNAs) targeting viral sequences (Tassetto et al., 2017). These siRNAs, secreted in exosome-like vesicles of immune cells, may represent the source of information storage. Furthermore, as TGIP requires the transfer of the information from the primary challenge to the offspring, the integration of viral elements into the genome through recombination with specific classes of retrotransposons and their organization into large loci of endogenous viral elements (EVEs) may represent a reservoir of immune memory in *Aedes aegypti* (Whitfield et al., 2017). Similar processes are unknown in *T. molitor*, which was also not reported to exhibit immune priming against virus infection.

Epigenetic reprogramming was also proposed as an important process to support within and trans-generational immune priming (Ottaviani, 2015). Epigenetic reprogramming of immune cells could be achieved through remodeling of DNA methylation patterns, changes in histone marks, modifications of chromatin structure, or changes in miRNA or lncRNA expression patterns. While indications of transcriptomic changes involving enzymes that control DNA methylation and histone acetylation in *Galleria mellonella* have been documented (Heitmueller et al., 2017), experimental evidence failed to link epigenetic differences to immune priming, both within and across generations (Eggert et al., 2014; Norouzitallab et al., 2015). A recent study has investigated the occurrence of methylation from total DNA and RNA extraction in *T. molitor* subjected to either individual or trans-generational immune priming by the fungus *Metarhizium anisopliae* or the bacteria *Micrococcus lysodeikticus* (Castro-Vargas et al., 2017). No global changes in DNA methylation resulting from either within or across generation immune priming were detected. However, whether DNA methylation was affecting smaller relevant portions of the genomic DNA, for instance, targeting the regulatory regions of a restricted number of genes was not investigated. In addition, a low proportion of RNA methylation results from individual immune priming, indicating that RNA methylation could be involved in the process. However, no such change results from TGIP. Further study is needed to identify the types of RNA involved in methylation and their implication in the individual priming process.

The involvement of microRNA, lncRNA, and changes in chromatin structure has not been investigated in TGIP, despite their involvement in invertebrate immunity and host-pathogen interactions (Asgari, 2013). In *G. mellonella,* experimental selection for resistance to *Bacillus thuringiensis* resulted in trans-generational modification of acetylation of specific histones, DNA methylation, and transcription of genes encoding the enzymatic writers and erasers of these epigenetic mechanisms (Mukherjee et al., 2017). Hence, considering the prominent role of epigenetics in many trans-generational adaptation processes in animals and its implication in the modulation of several immune response pathways, its involvement in immune priming, especially in TGIP, might be a promising avenue to explore in greater depth.

Immune priming, either within or across generation, is beneficial by enhancing individual immune protection against repeated infections. However, this process likely exerts energy-related costs that would constrain the expression of other important functions. These costs might be bearable upon high risks of repeated infection but could be heavy when re-infection is unlikely (Tate, 2017). The cost of individual immune priming includes the cost of the initial immune response, upon a primary contact with the pathogen, and the additional cost of keeping the immune system upregulated for an extended period of time (*i.e.* memory), which are almost impossible to discriminate. The cost of TGIP is likely shared by both parents and offspring. On the one hand, parents, especially mothers, may support part of the cost of TGIP by producing and transferring immune substances to their eggs in addition to paying the usual immune activation costs resulting from the priming infection (Moret and Schmid-Hempel, 2000). For instance, bacterially immune-challenged females of *T. molitor* transiently endow a variable proportion of their eggs with antibacterial activity, which negatively correlates with female fecundity (Zanchi et al., 2012). Furthermore, the level of antibacterial activity found in eggs correlates negatively to that of their mother’s hemolymph, suggesting that mothers trade-off their own immunity against that of their eggs (Moreau et al., 2012). On the other hand, enhanced immunity in the offspring may compromise other important functions. TGIP enhances immunity in the offspring of *T. castaneum* and *T. molitor* at the expense of a prolonged larval development time (Roth et al., 2010; Zanchi et al., 2011; Dhinaut et al., 2018a). A prolonged larval development time increases the probability of mortality (Bell, 1980), especially in tenebrionid beetles, which exhibits cannibalism on juveniles (Ichikawa and Kurauchi, 2009). However, such a cost in *T. molitor* depends on the bacterial pathogen to which mothers were previously exposed, as larval development time of maternally primed offspring with Gram-positive bacteria was much shorter than maternally primed offspring with Gram-negative bacteria (Dhinaut et al., 2018a). As pathogens may vary in the selective pressure they impose on hosts, *T. molitor* may have evolved an optimal immune priming against the most pervasive and threatening range of pathogens it encounters. In other insects, primed offspring exhibit reduced fecundity at the adult stage (Trauer and Hilker, 2013) and reduced resistance to a different parasite type to which the mother was exposed (Sadd and Schmid-Hempel, 2009). Further investigations are needed to reveal whether *T. molitor* primed offspring are paying comparable costs to TGIP. These negative effects associated to TGIP may result from the offspring trading-off their immunity against other functions. Alternatively, they may be the consequence of a reduced parental investment per offspring resulting from the cost of the parental immune challenge. In this case, reduced parental investment into their progeny should be observed early in the offspring’s life. However, recent evidence showed that immune-challenged *T. molitor* females produced eggs with a stronger hatching success and that the resulting young larvae show enhanced survival to starvation within the first month post hatching (Dhinaut et al., 2018b), although they are known to exhibit a prolonged developmental time later on (Zanchi et al., 2011; Dhinaut et al., 2018a). This suggests that TGIP cost is likely to arise from offspring trade-offs and not from a reduced parental investment.

### Microbiota

The importance of characterizing the mealworm’s microbial community is proportional to the increased interest in this insect as a food source. Indeed, understanding the microbiota of insects that are used for consumption is an essential for identifying potential spoilage bacteria and food pathogens. The bacterial community from living, processed, and laboratory-reared mealworms reared for consumption indicated that their microbiota was dominated by Tenericutes, Firmicutes, and Proteobacteria (Jung et al., 2014; Stoops et al., 2016; Garofalo et al., 2017; Osimani et al., 2018). The gut-associated microbiota is an important mediator of host development and growth. For instance, microbiota-free mosquito larvae (axenic individuals) developmentally arrest due to the lack of bacteria-mediated hypoxia (Coon et al., 2014; Coon et al., 2017). Another striking example is the contribution of the gut-associated bacteria *Lactobacillus plantarum* to *Drosophila* growth (Storelli et al., 2011). The bacterium promotes protein assimilation from *Drosophila*’s diet, optimizing diet-derived amino acid levels in the hemolymph. This activates the target of rapamycin (TOR) signaling pathway, which triggers the insulin-like and ecdysone pathways that promote growth rate and reduce growth duration, respectively (Storelli et al., 2011). While no such intimate interaction has yet been described between *T. molitor* and its microbiota, axenic *T. molitor* experiences a change in digestive enzyme expression, supporting the hypothesis that associated microbes are involved in the insect’s physiological homeostasis. Especially, the microbiota may help the mealworm to defend against the detrimental effects of food-derived toxic compounds such plant-derived glucoside salicin (Genta et al., 2006). In addition, microbe-free *T. molitor* does not produce pentadecene (Genta et al., 2006), a volatile that functions as a defensive secretion against predators in *T. castaneum* (Arnaud et al., 2002).

As previously mentioned, another growing interest for *T. molitor* concerns its ability to digest polystyrene foam. While polystyrene foam decreases *T. molitor* fecundity (Nukmal et al., 2018), the insect can fully develop using the plastic as its primary source of food. This makes the insect a relevant alternative to recycle polystyrene. Interestingly, when the mealworm’s microbiota is disrupted following an antibiotic treatment, the insect loses its ability to digest polystyrene, indicating that its associated microbes play a crucial role in the digestion process (Yang et al., 2015). Especially, the bacterium *Exiguobacterium* sp. (Firmicutes) was isolated from the midgut of mealworms and was demonstrated to degrade the polystyrene *in vitro* (Yang et al., 2015). This shows that specific members of the microbial community confer the mealworm its ability to digest polystyrene. Hence, targeting the microbial community of *T. molitor* could help to boost its polystyrene digestion efficiency. This could be achieved *via* isolation of bacteria originating from the mealworm microbiota that would be genetically engineered to produce polystyrene-degrading enzymes.

In addition to supporting insect growth, indigenous microbes can mediate the development and function of their host immune system. In tsetse flies, microbe-free adults exhibit a severely compromised immune system that is characterized by a significantly depleted population of hemocytes (Weiss et al., 2011). Interestingly, larval *Drosophila’s* indigenous microbiota regulates orthologous hematopoietic pathways in their host (Benoit et al., 2017). These examples demonstrate the intricate impact that the microbiota plays on host immune development. Such an association between *T. molitor* and its microbiota has yet to be investigated. Nevertheless, the enhanced immune response conferred by oral priming and TGIP demonstrates that immune mechanisms are adjustable according to *T. molitor* interaction with microbes. Also, it was suggested that microbiota influences oral priming in the red flour beetle *T. castaneum* (Futo et al., 2016). Hence, exploring whether *T. molitor*’s microbiota could influence TGIP would be a major milestone to understand the molecular mechanisms underlying this process.

The microbiota of *T. molitor* includes microbial taxa that may be pathogenic for human and animals, such as *Enterobacteriaceae*, *Streptococcaceae*, and *Enterococcaceae* (Jung et al., 2014; Stoops et al., 2016; Garofalo et al., 2017; Osimani et al., 2018). Methods used to process mealworm larvae for food, such as pulverization, could allow microbes to be released from the insect gut, which, if not eliminated, may be hazardous to livestock and human consumers (Klunder et al., 2012; Megido et al., 2016; Stoops et al., 2016; Garofalo et al., 2017; Grau et al., 2017). Thus, the presence of pathogens in mealworms would serve as an impediment to the use of this insect as a source of human and animal food. How the immune system could cope with controlling pathogens or modifying the microbiota structure is still an open question. However, the activation of the immune system through the production of antimicrobial peptides before processing the larvae could help to prevent an unwanted microbial community growing within insect-processed products. Indeed, insect antimicrobial peptides are active against a large range of microbes (Bulet et al., 1999). Immune priming could be used to trigger the long-lasting production of antimicrobial peptides in the hemolymph of the insect (Dhinaut et al., 2018a) prior to processing mealworm larvae. In consequence, primed insects would incorporate antimicrobial peptides that prevent unwanted microbes from contaminating the mealworm-derived food and feed.

## Promoting and Controlling *T. Molitor* Populations

*T. molitor* is increasing being recognized as an alternative source of protein-rich food with a low ecological footprint (Grau et al., 2017). Hence, insect farms have started to mass produced mealworms despite the risk of an infection outbreak. Each group of insect pathogens presents unique biological characteristics for which a clear understanding of the interaction with the host is required to implement efficient control (Eilenberg et al., 2015). Nevertheless, controlling disease involves improving the insect’s first line of defense, with the aim being to reduce the probability of infection. In *T. molitor*, one promising approach involves enhancing the integrity of the cuticle so as to render adult insects more resistant (Figure 3). Indeed, darker beetles present a thicker cuticle that is less likely to be circumvented by external pathogens (Silva et al., 2016; Evison et al., 2017), and they present enhanced immune parameters, such as the PO activity and hemocyte concentration (Armitage and Siva-Jothy, 2005). As *T. molitor* population density positively correlates with cuticle darkness, we can expect that mass rearing conditions are already optimized for this parameter. Temperature and food can also influence the quality of the cuticle, as higher temperature and better access to the aromatic amino acid tyrosine increase the darkness of the cuticle (Prokkola et al., 2013; Evison et al., 2017). While supplementation of tyrosine could be easily achieved, increasing the rearing temperature would cause some deleterious effects on the insect immune system, which makes it a versatile parameter to account for.

Immune priming could be an asset for the mass production of healthy insects while keeping in mind the deleterious effects on other traits of the offspring. However, all the studies investigating immune priming in *T. molitor* used systemic injection of immune elicitors to obtain primed insects. This may prove difficult in mass-reared insects due to their large number. If *T. molitor* could be orally primed like *T. castaneum* (Knorr et al., 2015), the provision of inactivated bacterial materials in the food should be valuable to prevent disease outbreaks in the insect cultures (Grau et al., 2017). Alternatively, managing individually the parental line to enhance offspring immune parameters would be an easier task than trying to apply a method to each mass-produced insect. TGIP or a cold shock experienced by the parents could potentially improve the immunity of the offspring (Eggert et al., 2015). Hence, applying such stress only to insects destined for reproduction could enhance the overall immunity of the colony (Figure 5).

Immune priming may also have strong implications for the control of populations of pest insects, such as *T. molitor*, using microbial bio-insecticides. Indeed, when failing to kill the insects, biocontrol agents may subsequently enhance the insect resistance or tolerance, rendering their use less efficient over time. The control of unwanted populations might be even further complicated when, like in *T. molitor*, the priming response provides cross-specific protection that would impair the efficiency of control strategies using different microbial pathogens. Furthermore, insects may not necessarily need to suffer from the infection by the pathogen to become primed, as the consumption of dead bacteria in the food could be sufficient to prime them, as shown for *T. castaneum* (Knorr et al., 2015). Such an infection-free priming process may keep the insects vigorous enough to maintain prolific reproduction while becoming more immunocompetent.

Optimizing the production of insects such as *T. molitor* would allow other uses, such as producing pharmaceuticals or using them for de-pollution purposes. Interest in using insect AMPs as an alternative to antibiotics in livestock production has been growing over the past decade (Li et al., 2014; Wang et al., 2016). Given the broad spectrum activity exhibited by insect AMPs, their production in heterologous systems can reveal difficult. However, *T. molitor’s* large mass, in conjunction with the ability to mass produce the insect, makes it useful for generating AMPs *via* immune stimulation during the rearing process. Because the purification of AMPs could prove difficult, the use of a whole insect extract could be a viable alternative.

*T. molitor* is especially relevant for its ability to digest polystyrene foam. It would be highly beneficial to optimize production of the insect, in combination with improving its digestive capacity via microbiota manipulation, as an alternative to conventional polystyrene recycling schemes. Fundamentally, *T. molitor* and its associated microbes are highly suited candidates for investigating microbe-driven impacts on insect development, with a special focus on the immune system maturation. Understanding the molecular dialog between the insect and its microbiota opens up the possibility for exploiting this interaction as a target for pest control strategies to undermine the insect defenses, or, on the contrary, for enhancing the insect’s defenses to optimize its application as a beneficial resource.

Recently, the insect immune system has been studied in the context of developing new tools for insect pest control. A proposed approach consists on using RNA interference (RNAi) to target genes that are crucial for the survival of insects (Baum et al., 2007). RNAi uses homologous double-stranded RNA (dsRNA) to downregulate specific mRNAs, which, for the purpose of pest control, would target genes leading to a severe decrease in fitness. This method was demonstrated successful on insects that feed on plants expressing hairpin dsRNA constructs, or on crops sprayed with dsRNA (Baum et al., 2007; Mao et al., 2007; Whyard et al., 2009; Huvenne and Smagghe, 2010). Recently, this method was applied to downregulate the expression of genes involved in *T. castaneum* Toll signaling pathway, especially focusing the pathway regulator *cactus* and its interacting genes (Bingsohn et al., 2017). RNAi-driven knockdown of *cactus* leads to the death of the insect, validating the relevance of targeting this gene as a novel control method. However, as *cactus*, and more globally the Toll pathway, is required for dorsoventral patterning in *Drosophila melanogaster* (Belvin and Anderson, 1996), the observed effect on *T. castaneum* may be due to a disruption in developmental processes rather than immune-related functions. Using such methods also brings the issue of collateral damage due to an unspecific effect of the RNAi, which could target conserved genes across different species that reside in the target insect’s environment. As developmental genes are usually highly conserved across taxa, targeting genes specifically involved in the immune system of an organism under infection could prevent such an effect. Knockdown of immune genes could be combined with the use of *T. molitor* parasites such as the Apicomplexan gregarines (Harry, 1967; Rodriguez et al., 2007) or the ectoparasitoid *Scleroderma guani* (Zhu et al., 2013) to successfully control the pest (Figure 4).

## Author Contributions

AV and YM conceived the ideas. AV, CJ, TR, and YM performed the literature search and contributed to the writing of the manuscript.

### Conflict of Interest Statement

The authors declare that the research was conducted in the absence of any commercial or financial relationships that could be construed as a potential conflict of interest.
